# Analysis of osseodensification with two drill systems versus conventional technique for dental implants –A paired comparative ex vivo study

**DOI:** 10.1371/journal.pone.0338078

**Published:** 2026-01-05

**Authors:** Rafaela Regina de Lima, Lara Rúbia Marques Nascimento, Barbara Magalhães Figueiredo Dias, Dhelfeson Willya Douglas-de-Oliveira, Rodrigo Richard da Silveira, Frederico Santos Lages

**Affiliations:** 1 Department of Restorative Dentistry, Faculty of Dentistry, Federal University of Minas Gerais, Belo Horizonte, Minas Gerais, Brazil; 2 Department of Dentistry, Federal University of Vales do Jequitinhonha e Mucuri, JK Campus, Diamantina, Minas Gerais, Brazil; Universidade de Trás-os-Montes e Alto Douro: Universidade de Tras-os-Montes e Alto Douro, PORTUGAL

## Abstract

**Objective:**

To compare conventional and osseodensification techniques assessing insertion/removal torque, stability (ISQ), maximum temperature, and bone structural alterations, as well as evaluating the performance of osseodensification with two bur systems: Densah®/Versah and Bone Reamer Drills®/WF.

**Methods:**

Sixteen bovine ribs were prepared into standardized samples. Each bone block received three osteotomies using different techniques following manufacturers’ protocols: G1 – conventional burs; G2 – osseodensification with Densah®/Versah burs (VS); G3 – osseodensification with Bone Reamer Drills®/WF burs. Osteotomies were performed under irrigation with 0.9% saline at 4–6 °C. Heat generation was recorded with a thermocouple; torque was measured at implant insertion and removal; ISQ was obtained after implant insertion using Osstell ISQ positioned 2 mm from the SmartPeg at a 45º angle. The burs were weighed before and after perforations and analyzed by scanning electron microscopy (SEM). Bone samples also underwent SEM analysis after implant removal.

**Results:**

The osseodensification groups exhibited increased insertion and removal torque values and bone compaction at the implant interface was observed. There was no significant increases in ISQ or maximum temperature compared to conventional drilling. No significant mass loss was observed in either systems.

**Conclusion:**

Bone instrumentation with both osseodensification kits improved primary implant stability, demonstrating higher insertion and removal torque values than conventional drilling in type IV bone. No significant ISQ and temperature differences were found. SEM revealed compacted bone at the implant interface in the osseodensification groups. No significant mass changes were observed.

**Clinical relevance:**

Results suggest that both techniques are safe and effective for clinical recommendation.

## 1. Introduction

The clinical success of implant therapy is multifactorial and one of the most relevant principles involves achieving adequate primary stability, which may vary according to the patient’s oral biological conditions [1–4]. Achieving primary stability relates to atraumatic surgical tissue preparation, which aims at a better healing response, avoiding trans- and postoperative complications, such as pain, edema, hemorrhage and early implant loss [5–9]. Acknowledging the importance of surgical steps, several researchers have been improving bone site preparation techniques, with recent attention to the osseodensification technique, in order to develop a condensed autograft around the implant [5,10].

Osseodensification is a technique for compacting bone microparticles, generated in the surgical drilling protocol in the alveolar bone wall [11]. The immediate contact of the implant with the compacted microparticles favors more efficient osseointegration due to the nucleation of osteoblasts in this region, which is associated with increased primary stability as a result of the physical interlocking [5]. Densifying burs have demonstrated the ability to drill the bone mineral tissue to the depth of the intended osteotomy when used in a clockwise cutting direction, as well as promoting the densification of the recipient alveolus when used in a counterclockwise direction [12,13].

Different systems present specific standards that guide the implantologist in the surgical and rehabilitation procedure [14,15]. The conventional technique for preparing the implant site differs according to bone availability, macrogeometry and the implant system chosen for rehabilitation [16]. Contrary to the osseodensification technique that establishes the surgical bed without excavation, the conventional technique uses burs with varying diameters to promote controlled and specific surgical access for each type of dental implant [14,16].

As a center of excellence in the dental science field, the Brazilian market develops burs for the osseodensification technique that still requires mechanical-physical investigations to allow their recommendation in Implantology, allowing the safe execution of drilling protocols with predictable and satisfactory results for patients [17]. Thus, it is necessary to establish if these new materials can offer competitiveness to the conventional technique based on their in vitro behavior. In this scenario, the objective of this study is to compare conventional and osseodensification with burs techniques based on insertion and removal torque values, stability coefficient, maximum temperature and structural changes; in addition to assessing the performance of osseodensification associated with two different bur systems – the widely used reference kit (Densah®/Versah) and a Brazilian-made kit (Bone Reamer Drills®/WF). The null hypothesis is that there is no difference between the results of the conventional evaluation techniques and osseodensification with burs.

## 2. Materials and methods

### 2.1. Sample selection and preparation

Bovine ribs bones were purchased from a local slaughterhouse and stored in thermal containers between −18 and −20ºC to preserve the integrity and characteristics of the tissue [18].

The animals were not used exclusively for this study and the bones were obtained from discarded waste material. Since the bovine ribs were obtained post-slaughter from the local food market, the study was not submitted to the animal ethics committee [19].

The bovine ribs were cleaned, the soft tissue removed and samples with approximate measurements of 50 mm (length) x 12 mm (width) x 30 mm (height) were prepared [18]. The bone blocks were allowed to thaw for a period of 05 hours at room temperature and were subsequently fixed in a bench vise to prevent movement during the tests [18].

### 2.2. Sample calculation

The OpenEpi v. 3.01 software was used to determine the value of n in studies comparing 2 means. A confidence level of 0.05 and statistical power of 0.80 were adopted. To evaluate the null hypothesis of this study, the minimum number of samples per group was determined to be 16, which was based on the similar methodology and objectives as described by Barberá-Millán et al. (2021) [20].

### 2.3. Osteotomy site preparation

The beds preparations were performed by the trained and calibrated examining researcher, following the manufacturers’ protocols, using a 20:1 reduction contra-angle (NSK Smax-SG20, Japan) coupled to the surgical electric micromotor with adjustable rotations per minute (rpm) and torque. Biomorse® 4x10mm implants (Bio Implante, Bauru) were installed in all groups with similar surface treatments and shapes.

The study was carried out on 16 bovine ribs. Three osteotomies were performed on each bone block using different techniques for dental implants, divided into three groups according to the technique and drilling bur: G1 - control group using the conventional technique; G2 - group using Densah®/Versah (VS) densification burs and G3 - group using Bone Reamer Drills®/WF densification burs. In all tests, the protocol recommended by the manufacturer was followed.

**Fig 1:**
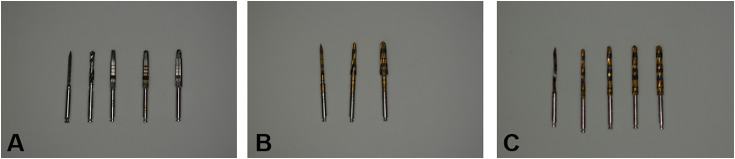
Images of the burs used in each group. **A)** Group 1. **B)** Group 2. **C)** Group 3.

For the control/conventional group (G1), osteotomy occurred in a clockwise direction in the following sequence of burs and their diameter: 1.8 mm spear-type, 2.2 mm helical, 2.8 mm conical, 3.2 mm conical and 3.6 mm conical. In the test group G2 (VS), the bed preparation was performed in a counterclockwise direction with an oscillating pumping movement, consisted on an oscillating movement of the bur in and out of the osteotomy, inducing slight pressure to the bone [12]. According to the manufacturer, in type IV bones, the final diameter of the osteotomy preparation should be driller with a Densah® bur with an average diameter 0.5–0.7 mm smaller than the average diameter of the implant. The VPLTT (pilot bur, only this one in a clockwise direction), VT1828 (2.3 mm), and VT2838 (3.3 mm) burs were used in sequence. In the G3 (WF) test group, the surgical site was prepared in a counterclockwise direction with an oscillating bombardment movement. The surgical drilling sequence was: helical lance 1.8 mm, BRD 2.2 mm, BRD 2.6 mm, BRD 3.0 mm and BRD 3.4 mm (Fig 1).

In all groups, and following the speed range recommended by the manufacturers, a speed of 1000 rpm was used. All osteotomies were performed with saline solution (0.9% sodium chloride) at a temperature between 4 °C and 6 °C, using the system integrated into the surgical motor, which directed the flow to the body of the burr. All the surgical procedures and tests were performed by a trained and calibrated researcher, and the recorded data were transferred to a table for analysis.

### 2.4. Temperature test

Heat generation was measured during osteotomy using a thermocouple, which is a direct method that uses a heat-sensitive probe connected to thermometers and/or computer software [21]. The probe insulation, recorded depth, sensor material, and other technical factors may influence its results [21]. The thermocouple records the local temperature and does not detect the overall thermal profile or heat leakage [21].

**Fig 2:**
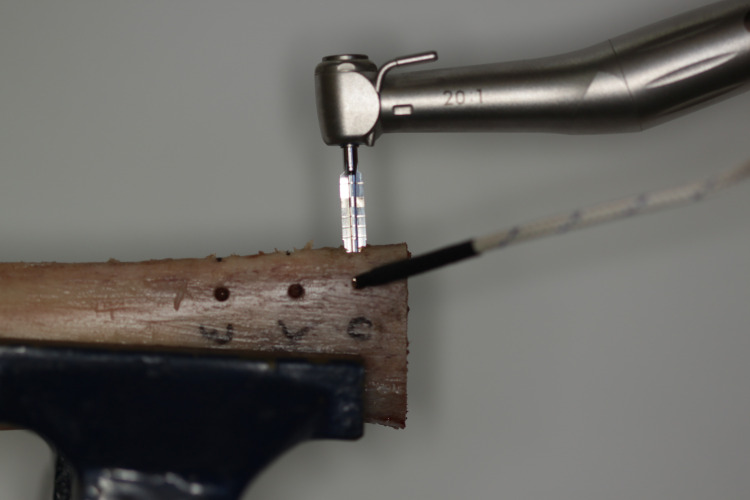
Location of the thermocouple probe during osteotomy for each group.

The thermocouple probe was inserted into the bone approximately 1 mm from the edge of the final diameter of the osteotomy [12] (Fig 2). To provide this distance, individual calculations were performed for each sample. The drilling depth was standardized at 5 mm – which corresponds to half the height of the implant used – measured from the point of drilling of the implant on the bone crest. For the width, the value adopted was half the total width of the bone sample minus 3 mm, of which 2 mm corresponds to half the diameter of the implant used, and 1 mm refers to the final distance of the thermocouple probe. All drillings for the thermocouple probe were performed with 2.2 mm lance-type and helical drills from the Biomorse® implant surgical kit (Bio Implante, Bauru).

**Fig 3:**
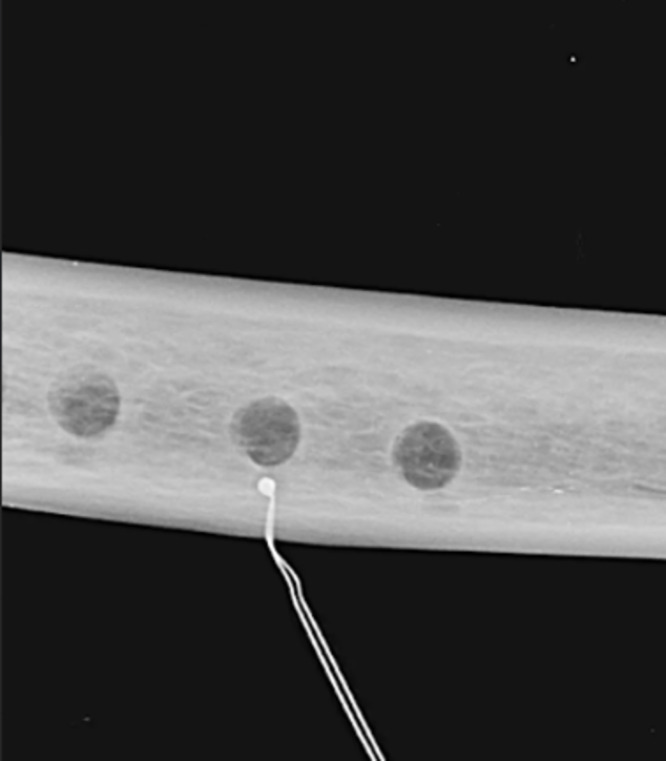
Radiograph highlighting the location of the thermocouple probe approximately 1 mm from the final edge of the osteotomy.

Radiographs were acquired to assess the position of the thermocouple probe tip (Fig 3) and the maximum temperatures were recorded during the use of each bur.

### 2.5. Insertion and removal torque tests

The torque during implant insertion was recorded at the time of their installation in the surgical site. All the torque values were recorded using a Lutron TQ8800 digital torque meter (Lutron TQ-8800, Taipei, Taiwan), with an accuracy of 0.1 Newton centimeters (Ncm) coupled to a computer.

After drilling, the three groups of implants were manually inserted into the beds using a manual torque wrench (Bio Implante, Bauru) until 1 mm from their final position in the bone. Afterwards, a digital insertion wrench was attached to a high-precision calibrated digital torque wrench (Lutron TQ-8800, Taipei, Taiwan) and the insertion torque was measured when it reached its final position. For statistical analysis, the maximum insertion torque value was considered the insertion torque. The removal torque was recorded continuously, and the highest value required for the implant to unscrew from the bone block was recorded.

### 2.6. Resonance Frequency Analysis (RFA)

**Fig 4:**
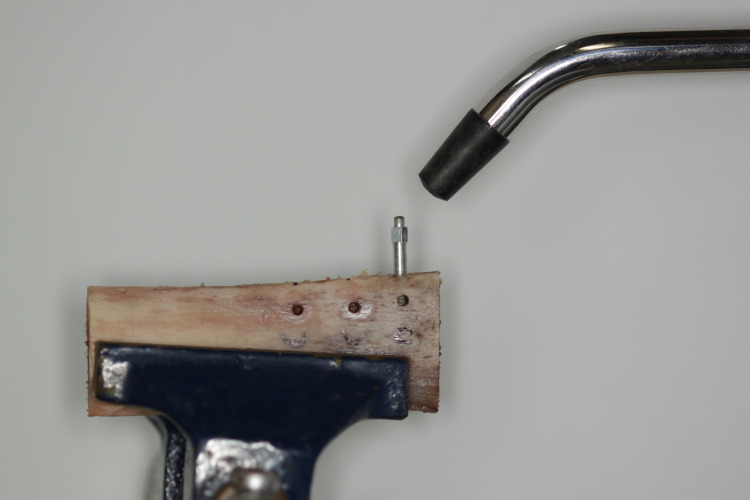
Measurement of the resonance frequency of the implants.

A SmartPeg type 01 transducer (Osstell®, Göteborg, Sweden) was used for each implant, and four measurements were performed, one on each face, resulting in a total of four records per implant. The measurements were performed consecutively, following the order of the distal, vestibular, mesial, and lingual faces of each implant. Using the Osstell®, the Implant Stability Quotient (ISQ) values of the implants were measured in all groups (Fig 4).

For recording, the transducer was screwed directly onto the implant, with a torque of 5–8 N using a specific plastic screwdriver accordingby the manufacturer’s instructions. The recording was obtained by placing the Osstell ISQ probe approximately 2 mm from the SmartPeg at an angle of 45º relative to the largest axis of the implant.

### 2.7. Scanning Electron Microscopy (SEM)

The scanning of the surfaces of the burs and bone samples was performed using scanning electron microscopy. This approach allowed the evaluation of any structural changes, such as wear on the cutters, cracks, splits, or gaps, in addition to the quality of bone compaction in the area where osseointegration would occur.

The bur samples were subjected to SEM before and after drilling. The burs were cleaned with isopropyl alcohol (99.8%) after drilling and no preparation was necessary.

The bone samples were subjected to SEM after removal of the implants from the bone beds. The samples were sectioned using a precision saw with a diamond disk, cleaned, fixed, dehydrated, mounted on a specific support, and metallized, according to the preparation recommended by the Microscopy Center of the Federal University of Minas Gerais (CM-UFMG).

All SEM tests were performed by CM-UFMG using a Scanning Electron Microscope (FEI Quanta 200 FEG, FEI COMPANY, Oregon, USA) with magnifications of 40x, 100x, 500x and 1000x. The surface differences between the two burs (Versah and WF), their wear and the surgical bed after removal of the implant were comparatively and qualitatively assessed.

### 2.8. Mass loss measurement for the osseodensification technique

The milling bur samples from groups G2 and G3 were weighed on an analytical balance (Marte® AL500, São Paulo, Brazil) with an accuracy of 0.001 g (1 mg). The milling bur samples were weighed before and after drilling and cleaned with 99.8% pure isopropyl alcohol.

## 3. Statistical analysis

For insertion and removal torque tests, implant stability coefficient, and maximum temperature, descriptive analysis were performed to obtain the mean and standard deviation, as well as the Shapiro-Wilk normality test. The repeated measures ANOVA test was applied due to the independent samples with parametric distribution, followed by Bonferroni post-hoc. A significance level of 5% (p < 0.05) was adopted. To analyze the variation in cutter mass, descriptive analyses were performed to obtain the mean and standard deviation. The Shapiro-Wilk normality test and paired t-test were applied. A significance level of 5% (p < 0.05) was adopted. All assessments were performed using the Statistical Package for Social Sciences version 21 (IBM SPSS, Armonk, NY: IBM Corp).

## 4. Results

### 4.1. Insertion torque, removal torque, implant stability coefficient and bed maximum temperature

There were statistically significant differences among the mean insertion torques of the conventional, VS, and WF groups (*p =* 0.007). Groups 2 and 3 showed higher mean insertion torque values when compared to group 1, with values of 77.62 Ncm (+/-31.6); 95.25 Ncm (+/-45.8) and 61.25 Ncm (+/- 29.6) respectively. In the intergroup analysis, statistically significant differences were identified between the conventional x VS (*p =* 0.018); conventional x WF (*p =* 0.004) and WF x VS (*p =* 0.016) groups.

There were statistically significant differences in the removal torque values among the conventional, VS, and WF groups (*p =* 0.008). Groups 2 and 3 showed higher mean removal torque values compared to Group 1, with values of 69.62 Ncm (+/- 35.6); 89.62 Ncm (+/- 48.4) and 58.18 Ncm (+/- 32.7) respectively. In the intergroup analysis, statistically significant differences were identified between the conventional x VS (*p =* 0.043); conventional x WF (*p =* 0.001) and WF x VS (*p =* 0.009) groups (Table 1).

**Table 1:** Results of the insertion torque and removal torque tests.

	Conventional	VS	WF		
	Mean (SD)	Mean (SD)	Mean (SD)	p	Post-hoc
Insertion torque	61.25 (29.6)	77.62 (31.6)	95.25 (45.8)	0.007	Conv. *x* VS: 0.018 Conv. *x* WF: 0.004 WF *x* VS: 0.016
Removal torque	58.18 (32.7)	69.62 (35.6)	89.62 (48.4)	0.008	Conv. *x* VS: 0.043 Conv. *x* WF: 0.001 WF *x* VS: 0.009

There were no statistically significant relationship between the stability coefficients recorded in the conventional, VS and WF groups (*p =* 0.157). The mean resonance frequency values for the conventional group were ISQ = 79.42 (+/- 6.81); VS ISQ = 82.40 (+/- 4.03) and WF ISQ = 80.68 (+/- 5.00) (Table 2). The mean maximum temperatures of the conventional group were 27.81ºC (+/- 2.94); 30.62ºC (+/- 4.61) in the VS group and 28.37ºC (+/- 2.68) in the WF group (Table 3). There was no statistically significant difference between the maximum temperature for the three groups (*p =* 0.087).

**Table 2:** Comparison of implant stability coefficient (ISQ).

	Conventional	VS	WF	
	Mean (SD)	Mean (SD)	Mean (SD)	p
Implant stability coefficient (ISQ)	79.42 (6.81)	82.40 (4.03)	80.68 (5.00)	0.157

**Table 3:** Comparison of maximum temperature (ºC) obtained during bed preparation.

	Conventional	VS	WF	
	Mean (SD)	Mean (SD)	Mean (SD)	P
Maximum temperature (ºC)	27.81 (2.94)	30.62 (4.61)	28.37 (2.68)	0.087

### 4.2. Comparison of the mass of Densah® (Versah) and Bone Reamer Drills® (WF) burs before and after drilling the surgical beds

There were no statistically significant differences between the mean values of the mass of the burs of groups G2 (Densah®, Versah) (*p =* 0.667) and G3 (Bone Reamer Drills®, WF) (*p =* 0.208) before and after drilling. The mean mass values were 0.333 mg (+/- 1.155 mg) for Densah® burs; and 0.600 mg (+/- 0.894 mg) for Bone Reamer Drills® burs (Table 4).

**Table 4:** Comparison of mass (mg) of Densah® (VS) and Bone Reamer Drills® (WF) burs.

	Mass (mg)		
	Mean (SD)	Mean (SD)	p
VS	0.333 (1.155)	0.600 (0.894)	0.667
WF	69.62 (35.6)	89.62 (48.4)	0.208

### 4.3. Scanning Electron Microscopy – SEM

#### 4.3.1. Comparison between Densah® (Versah) and Bone Reamer Drills® (WF).

Images of the initial (pilot) burs and the final burs recommended by each system’s manufacturer were obtained before and after drilling to compare the structural changes in the drills throughout the experiment. It was not possible to evaluate wear on all cutting surfaces of the drills, as only one cutting surface of each bur was randomly selected based on the drill’s orientation relative to the SEM electron beam. However, none of the drills exhibited gross deformation on their visible surfaces at any stage of the experiment.

**Fig 5:**
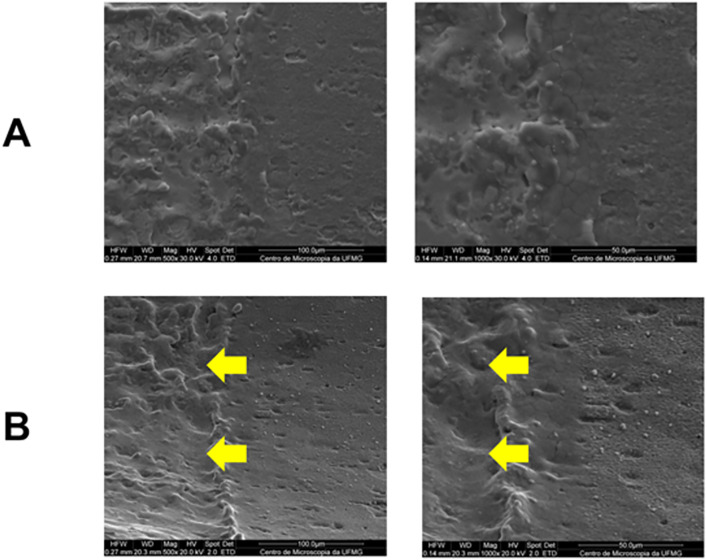
Scanning electron microscopy images of the interface region between the active tip and the body of the VT2838 bur. Images with magnifications of 500x and 1000x, respectively. **A)** Images obtained before bed preparation; **B)** Images obtained after bed preparation. A slight structural loss can be observed in the areas indicated by arrows.

The final Densah® drill (VT2838) showed slight structural loss at 500× and 1000 × magnifications in the interface region between the active tip and the body (areas indicated by arrows) (Fig 5). The structural difference between the active tip and the body suggests the presence of a surface treatment applied to the active tip.

**Fig 6:**
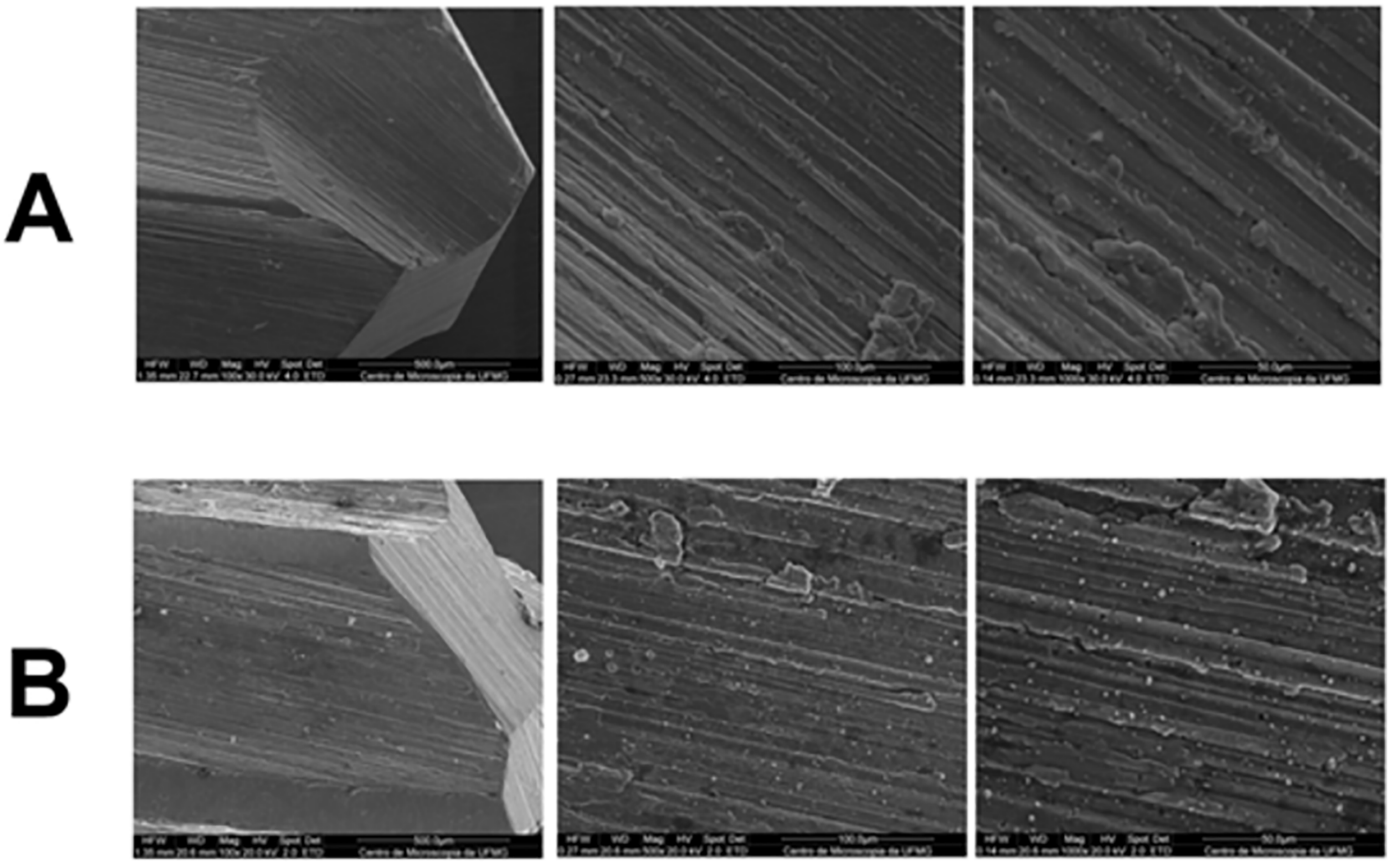
Scanning electron microscopy images of the initial bur from the Bone Reamer Drills® (WF) system. Images with magnification of 100x, 500x and 1000x respectively. **A)** Images obtained before bed preparations; **B)** Images obtained after bed preparations. Slight visible wear and areas of deformation can be observed at the active tips.

**Fig 7:**
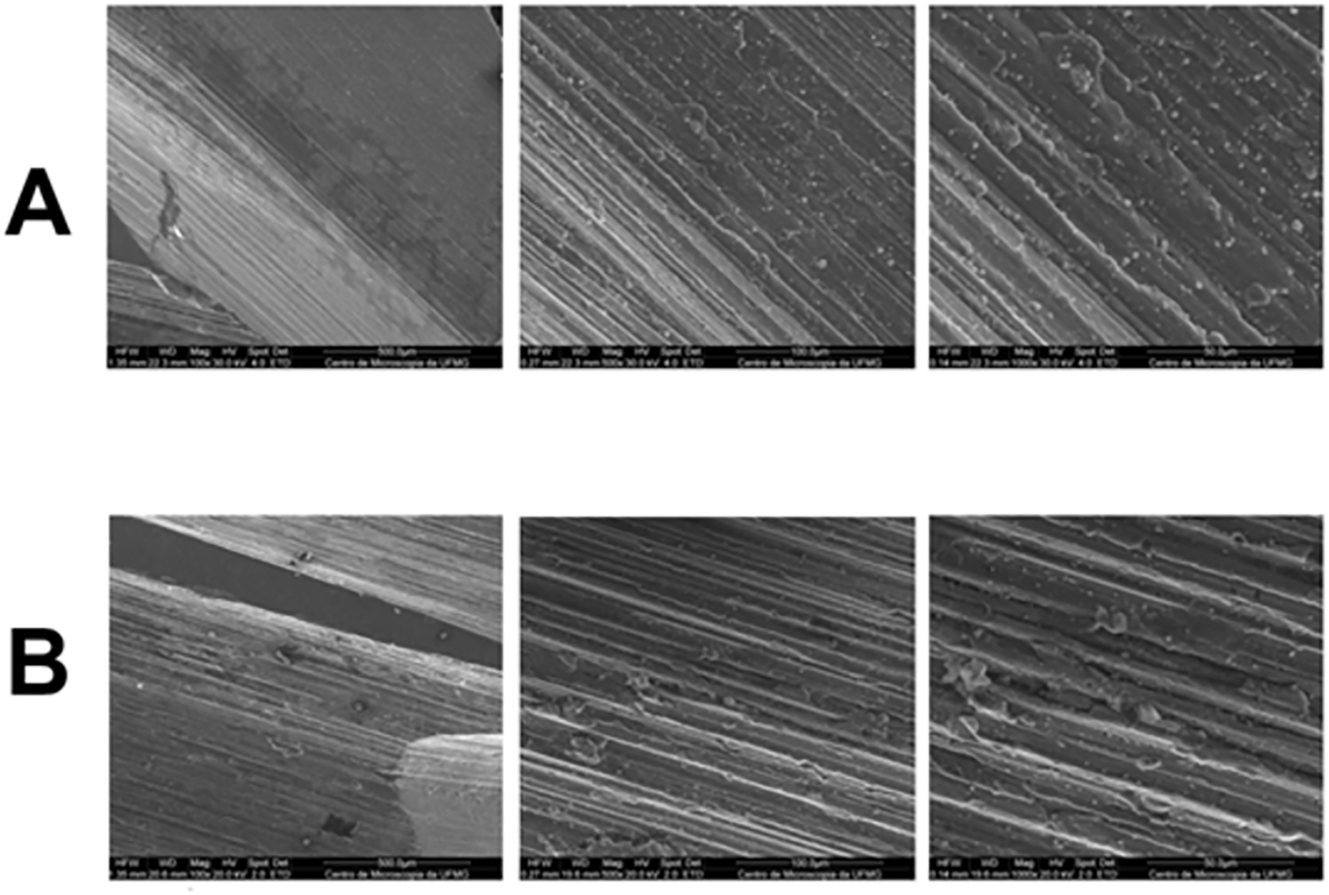
Scanning electron microscopy images of the final bur from the Bone Reamer Drills® (WF) system. Images with magnification of 100x, 500x and 1000x respectively. **A)** Images obtained before bed preparations; **B)** Images obtained after bed preparations. Slight visible wear and localized deformation can be observed at the active tips.

The pilot bur (helical lance 1.8 mm) (Fig 6) and the final bur (BRD 3.4 mm) (Fig 7) from the Bone Reamer Drills® (WF) system showed evidence of surface irregularities along their active tips, visible under SEM both before and after the experiment, suggesting areas of wear or possible plastic deformation. The WF kit burs exhibited more extensive wear compared with the Densah® burs, with a more uniform pattern of surface alteration after use.

**Fig 8:**
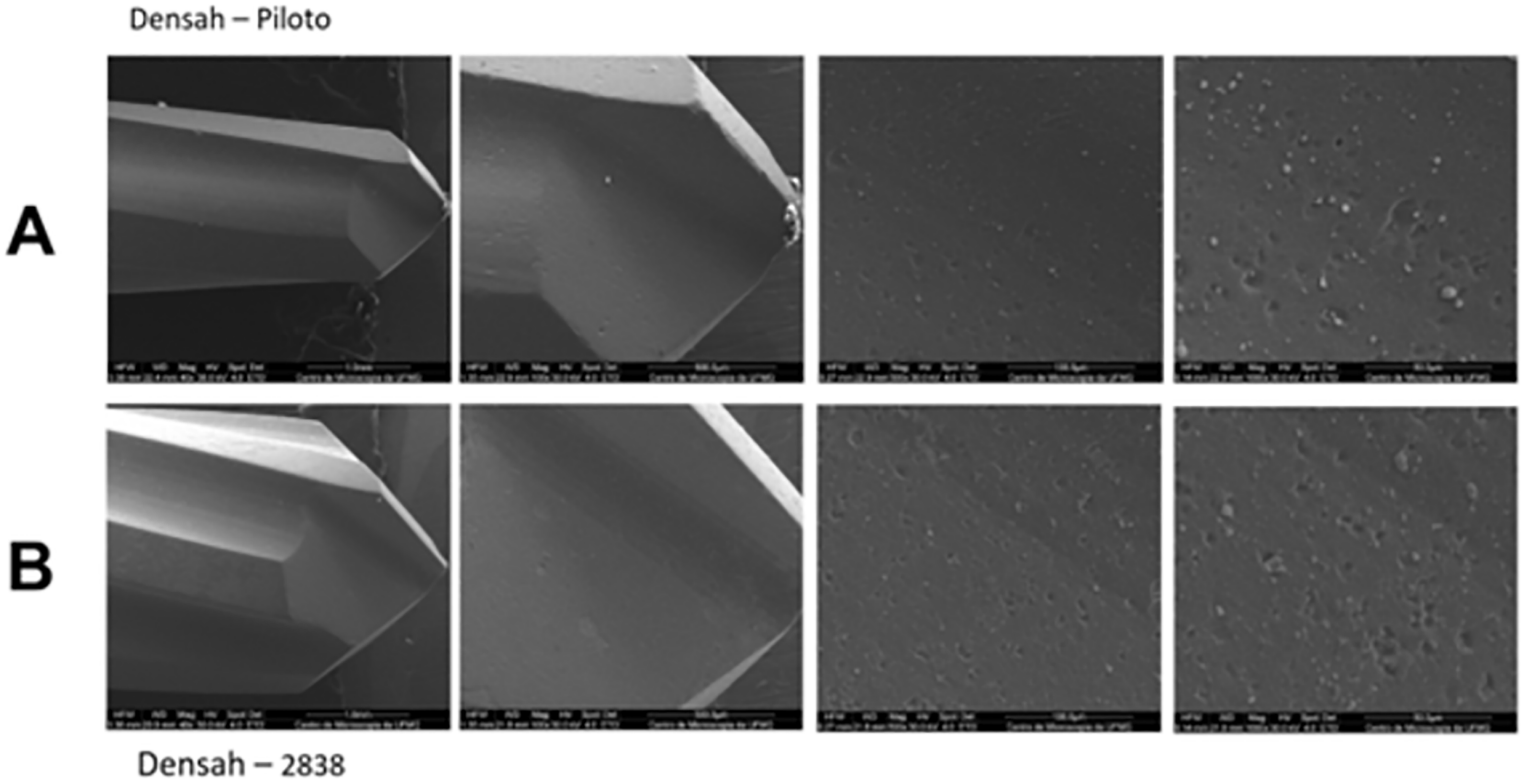
Scanning electron microscopy images of the surface of Densah® VPLTT and VT2838 cutters. Images with magnification 40x, 100x, 500x and 1000x respectively. A) images of the surface of the VPLTT pilot milling cutter. B) images of the surface of the VT2838 final milling cutter.

SEM magnification revealed differences in the manufacturing process between the two instruments. The Densah® VPLTT and VT2838 burs exhibited smooth surfaces, particularly at their active tips, suggesting the presence of a coating or surface treatment applied during manufacturing (Fig 8).

**Fig 9:**
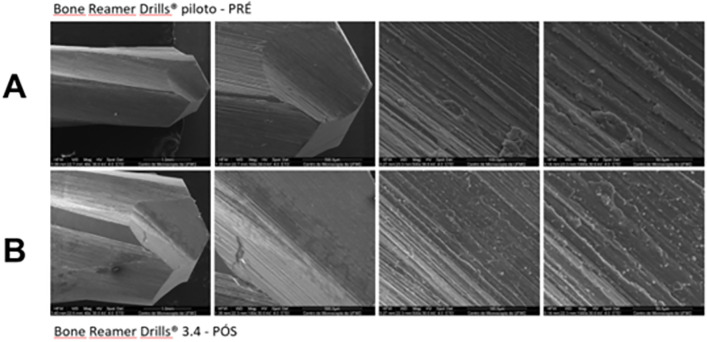
Scanning electron microscopy images of the surface of Bone Reamer Drills® helical 1.8 mm. Images with magnification 40x, 100x, 500x and 1000x respectively. A) images of the surface of the 1.8 mm pilot milling cutter. B) images of the surface of the 3.4 mm BRD final milling cutter.

The Bone Reamer Drills® pilot and final drills (1.8 mm helical lance and 3,4 mm BRD, respectively) exhibited visible machining lines across their entire examined surfaces (Fig 9), indicating a different manufacturing process compared to the Densah® drills.

#### 4.3.2. Comparison between conventional Biomorse® (Bio Implant) X Densah® (Versah) x Bone Reamer Drills® (WF) surgical sites.

**Fig 10:**
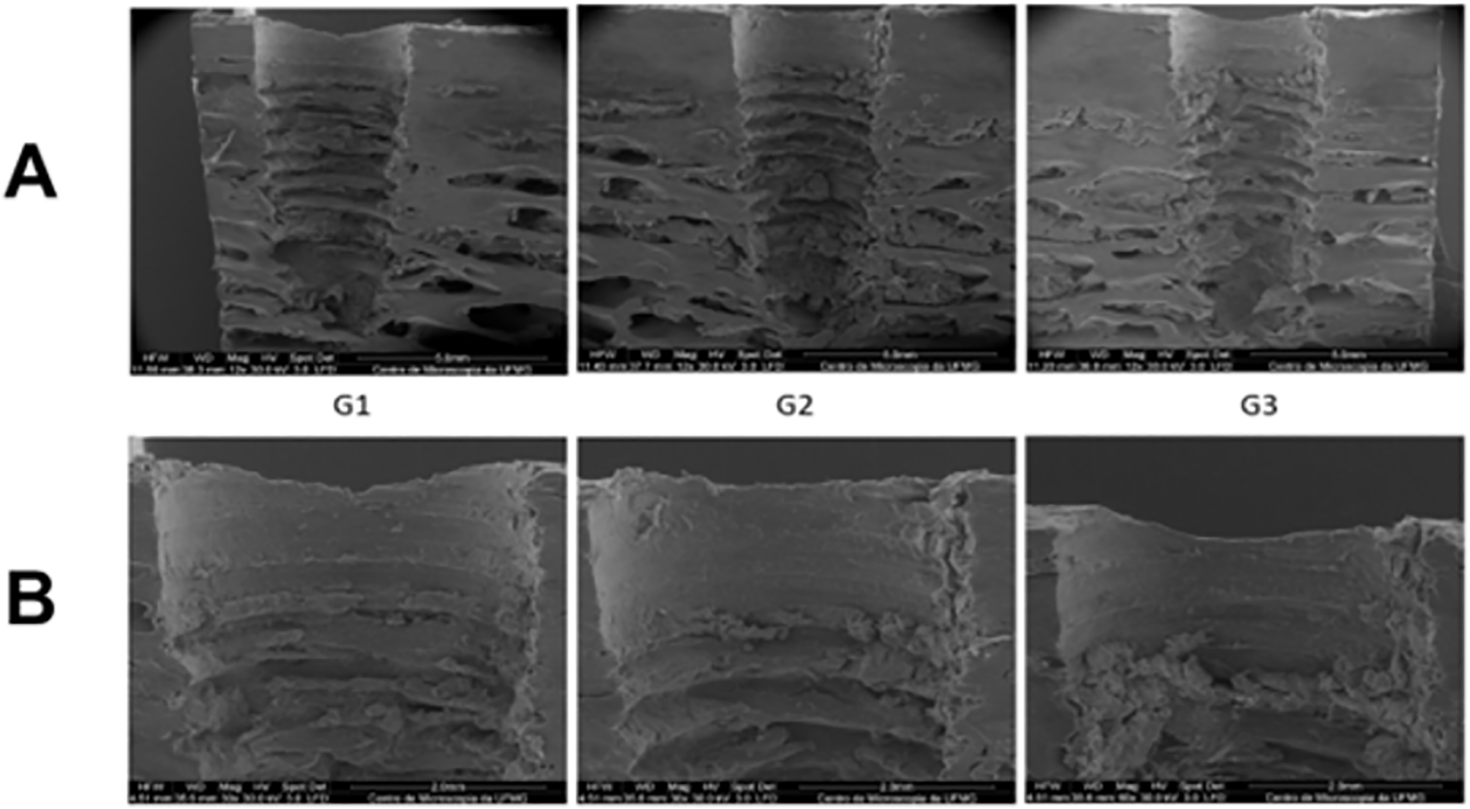
Scanning electron microscopy of the surgical beds of the three groups after implant removal. Images magnified at **A)** 12x and **B)** 30x.

Scanning electron microscopy revealed images of the surgical beds of the three groups (G1, G2 and G3) after implant removal from the same bovine rib sample (Fig 10) allowing comparison of the structural alterations that occurred in the bone throughout the experiment.

**Fig 11:**
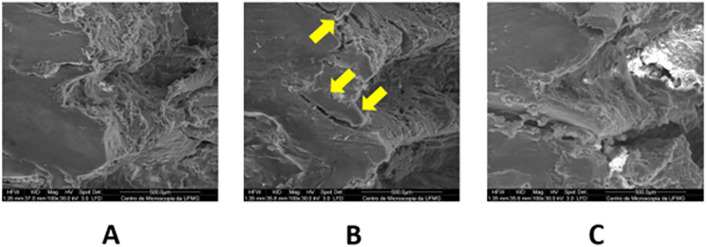
Scanning electron microscopy of the surgical beds of groups G1, G2 and G3. A) conventional group (G1). **B)** Versah group (G2). **C)** WF group (G3). Image at 100x magnification. Note the crack areas indicated by the arrows.

**Fig 12:**
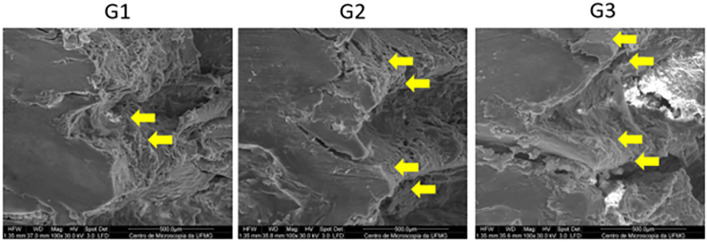
Scanning electron microscopy of the surgical beds of groups G1, G2 and G3. Images with 100x magnification. Note the absence of a compacted bone layer in G1 (arrowed area), while groups G2 and G3 display the presence of this layer.

Superficial structural changes in the bone tissue associated with the different drilling techniques were observed. Group G2 exhibited greater propagation of cracks (arrowed areas) (Fig 11). In addition, both G2 and G3 showed the presence of a compacted bone layer corresponding to the bone–implant interface. In contrast, this layer was not observed in G1 (arrowed area) (Fig 12).

## 5. Discussion

The osseodensification techniques resulted in higher insertion and removal torque values, as well as more pronounced structural changes in the bone bed. However, no increase in ISQ values or maximum temperature was observed when compared with the conventional technique. Furthermore, despite differences in the manufacturing processes between the two systems evaluated, no loss of mass was detected after use.

Compared to the conventional technique and similar to what has been reported in the literature, the present study found higher insertion torques in the osseodensification groups [12,20,22–24]. The osseodensification technique is capable of improving insertion torque by up to 24 Ncm in bones with low density due to the large amount of cancellous bone [25]. These results may be related to the condition of the bone bed in which the implant was inserted. The quantity and quality of bone present in the surgical bed directly influences the primary stability of the implant [26], considering it is largely determined by the friction generated during insertion of the implant into the intraoral bone [2]. In Groups G2 and G3, the pilot burs operate in a clockwise direction, removing bone from the osteotomy site, whereas the subsequent burs work in a counterclockwise direction, compacting the bone within the osteotomy without removing it. Thus, the higher torque values can be explained by the bone compaction around the implant promoted by the osseodensification technique, which, when properly performed, is capable of providing greater accumulation of bone tissue on the surface of the implanted screw [5,12]. Consequently, bone-implant contact increases and micromovement decreases, resulting in higher insertion torque due to enhanced primary stability. The final burs diameters used in groups G2 and G3 were comparable, indicating that the subinstrumentation effect was not a contributing factor in this experiment. Previous studies have shown that under-preparation, characterized by the use of smaller final burs diameters, increases insertion torque and primary stability of dental implants [27]. Therefore, since similar final diameters were employed in both groups, this variable did not influence the outcomes observed. On the other hand, the lower torque values in the conventional technique may be related to the osteotomy process, in which no bone residues remain on the walls of the surgical bed, representing a disadvantage compared to preparing the bed using the osseodensification technique.

Among the osseodensification groups, G3 showed even higher insertion torque values when compared to G2. High torque levels may be unfavorable due to the release of titanium particles associated with damage to the implant surface, which act as cofactors involved in bone loss around dental implants [28–30]. From an economic perspective, the G3 burs manufactured in Brazil are more affordable, favoring their use by a greater number of dental professionals. However, there are no studies available in the literature that allow for a better comparison of the results obtained.

The removal torque is a parameter used in scientific research to assess implant stability within the implant bed and is directly correlated with the percentage of bone volume surrounding the implant and the bone/implant contact (BIC) [31]. Consequently, the osseodensification technique has been shown to increase the secondary stability of the implant, as reflected by the removal torque values [11]. Although the aim of this study was not to assess the secondary stability of the implants, this research revealed increased removal torque values for the osseodensification groups compared to the conventional group, corroborating previous reports in the literature [12,22,23].

The Osstell® device is the main tool used to measure RFA values, ranging from 1 to 100, with 100 representing the highest stability value, referred to as the Implant Stability Quotient (ISQ) [32]. In the present study, there was no significant relationship between the stability coefficients recorded in the three groups (*p =* 0.157). Although these findings are similar to those of other studies [24,33,34], it is important to consider that different methodologies were employed among the studies mentioned.

Lages et al. (2017) [32] conducted a systematic review assessing whether the primary stability of dental implants could be evaluated using insertion torque and resonance frequency analysis. The researchers concluded that these are independent and non-comparable methods, and that the professional should select only one method for evaluation. In contrast, Cáceres et al. (2020) [22] found a positive relationship between ISQ and insertion torque, reporting higher values for both parameters in implants placed by the osseodensification technique, possibly due to the greater number of observations and the smaller number of comparative groups.

Several studies in the literature state that osseodensification improves the primary stability of implants in low-density bones when compared to the conventional technique, attributing the improved bone healing to the preservation of bone tissue and autografting of the local bone matrix along the osteotomy bed [12,20,23,25,35,36]. However, it is prudent to consider using the osseodensification technique in regions with denser bone, as the technique may not present the same benefits as those found in regions with more porous bones [34].

The temperature threshold for irreversible heat-induced bone damage ranging from 44–47°C when applied for 1 minute and measured approximately 0.5 mm from the implant [37]. Adequate irrigation is essential to prevent overheating during osteotomy, particularly when using the final drill diameter, and it is strongly recommended that the operator employ cooled irrigant and sharp drills to minimize the risk of thermal injury to the bone [38]. Additionally, Soldatos et al. (2022) also observed that the initial drills generate more heat, and the temperature variation decreases as the drill diameter increases, especially in conical drills [39].

The thermocouple probe was positioned about 1 mm from the edge of the final osteotomy diameter to avoid contact damage and accurately record temperature changes during site preparation [12,38]. In this study, identical speed and torque parameters were used for all groups. No statistically significant differences in maximum temperatures were observed among the three groups, and the results between G1 and G2 are consistent with previous findings in the literature [12,39,40].

The Densah® burr is capable of inducing compression movement in cancellous bone, resulting in controlled bone deformation due to its viscoelastic and viscoplastic characteristics [25]. In this study, it was possible to observe a layer of compacted bone around the bed of osteotomies performed using the osseodensification technique. It is suggested in the literature that the formation of a compacted bone layer around the bed of the osseodensification technique results in an increase in primary stability, secondary stability, and bone-implant contact (BIC) [11,24,25].

Morphological assessment of the surgical beds by microtomography revealed greater peri-implant bone volume in osseodensified beds compared with the conventional technique, indicating that trabecular condensation contributed to the increased bone volume, which favors the immediate stability of the implant through physical interlocking [24]. Similarly, Trisi *et al*. (2016) also reported a greater percentage of bone volume in beds prepared using the osseodensification technique, which was approximately 30% greater compared with the conventional technique [11].

Dental implant burs are reusable and widely adopted in clinical practice to perform osteotomies for dental implants. However, there are no clear guidelines regarding their longevity, and clinicians must determine their service life based on tactile perception of the force required to perform the osteotomy [38]. This decision regarding bur replacement may lead to premature replacement of the bur or prolonged use of a worn bur [41]. The ideal service life of a bur may fluctuate. Based on the technical report by Medical Data International (1999), Allsobrook et al. (2011) reported that a bur can be used in approximately 25 osteotomies, considering that the average number of implants placed per procedure is 2.5 implants, which diverges from most manufacturers’ recommendations, limiting reuse to 10 surgical procedures [38]. Reusing worn burs may cause significant damage to bone tissue, impairing osseointegration of the implant with the bone [42]. This study evaluated the wear of the initial and final burs using the osseodensification technique (G2 and G3). As in Allsobrook et al. (2011), it was not possible to compare the progressive wear on the active surfaces of the burs. Therefore, a single random drilling surface was analyzed during the microscopy sessions based on its orientation in the SEM [38]. Furthermore, a single specimen of each bur was for SEM analysis at different times, limiting the range of comparisons.

Bur wear is affected by the type of osteotomy required for each implant, as this influences the location on the bur where the bone is cut, resulting in variations that lead to different degrees of bur wear [41]. The initial (pilot) burs suffer greater wear than other burs used in osteotomy procedures for dental implants, requiring replacement after approximately 50 drillings [42]. Versah recommends replacing its Densah® burs after 12–20 osteotomies [43]. The present study employed the burs to perform 20 osteotomies. In the analyses, the pilot bur did not demonstrate major structural losses under SEM. The final bur (VT2838) presented an area of structural loss in the region of the active tip boundary and the bur body, suggesting the presence of a surface treatment on its active tip. There were no statistically significant differences between the masses of the burs before and after use.

According to WF (2025, p.10) [44], Bone Reamer Drills® burs are made of stainless steel coated with diamond-like carbon (DLC). This coating has biomedical applications as it leads to an increase in mechanical properties, providing increased resistance to corrosion and wear [45]. There is no information available on the number of reuse cycles that Bone Reamer Drills® can be subjected to before their replacement. Mendes *et al*. (2014) [45] found no significant differences in the mass of cutters with DLC coating after 10, 20, 30, and 40 drillings, although delamination of the coating was observed after multiple drillings. The latter reinforces our conclusions, since the burs did not exhibit structural losses in SEM and there were no statistically significant differences between the masses of the burs before and after the experiment. Although this study allows an objective evaluation of mechanical parameters comparing conventional and osseodensification techniques, as well as the performance of two different kits, its main limitation is that it was conducted in an in vitro scenario, which does not fully replicate the clinical environment Another limitation is the lack of studies on burs from group G3.

New research applying *in vivo* models and/or using Bone Reamer Drills® may provide important data to improve the safety and outcomes of dental implant treatments. Furthermore, single samples of a bone block and specimens of each drill were used for SEM evaluations, which may have limited broader comparisons. Further laboratory studies and analyses employing larger sample sizes are necessary to provide more information on the subject, enabling more effective tests to be performed, such as the Bone Area Fraction Occupancy (BAFO). However, our results suggest that osseodensification indeed forms a layer of compacted tissue around the surgical bed, favoring primary stability in bones considered to be of low density (type 4). Furthermore, future research should include a greater number of burs and drillings to verify the occurrence of significant structural changes and determine how frequently these changes occur.

## 6. Conclusion

Bone instrumentation using both osseodensification kits increased the primary stability of implants in type IV bone without causing a rise in temperature that could compromise bone viability, indicating that the technique is considered safe when adequate irrigation is applied. The wear observed on the drills was minimal, and SEM analysis revealed bone compaction at the implant–bone interface, suggesting an improvement in the local density of the implant bed.

These results indicate that the technique is promising for clinical use, although further studies are needed to evaluate its performance under clinical conditions, including the effects of irrigation, cleaning, and sterilization of the drills.
